# Supplementary material for: Pro- and anti-tumour activities of CD146/MCAM in breast cancer result from its heterogeneous expression and association with epithelial to mesenchymal transition
Source: Front Cell Dev Biol. 2023 Apr 17;11:1129015. doi: 10.3389/fcell.2023.1129015 (PMC10150653; doi:10.3389/fcell.2023.1129015)
Supplement: Supplementary file 1 [file DataSheet1.pdf]

Supplementary information

Mannion et al: Pro- and anti-tumour activities of CD146/MCAM in breast cancer result from its heterogeneous expression and association with epithelial to mesenchymal transition.

1. Supplementary Figures and Legends

References are those from the main manuscript.

Supplementary Figure 1

A) Verification of expression of ESR1 (oestrogen receptor), PGR1 (progesterone receptor), ERBB2 (HER2) and EPCAM genes in ER+ breast cancers, triple negative breast cancers (TNBC) and adjacent normal breast tissue from the study of Varley et al (37). The P values indicate a one-way ANOVA test to test for significant variance across the samples.

B) MCAM gene expression in breast cancer versus normal tissue as determined using the GENT2 database (39). Metadata from two different microarray platforms are indicated, with numbers of samples of tumour (red) and normal tissue (blue) indicated. ****P<0.0001 as determined by GENT2 using a two tailed Mann Whitney test.

Supplementary Figure 2.

Methylation of the MCAM promoter. A graphical representation of the MCAM gene on chromosome 11q23, highlighting exons, the transcriptional start site (TSS; red arrow) and a CpG island located from approximately -300 bp to +900 bp relative to the TSS. The precise sequence position of the CpG island on chromosome 11 is from positions 119317436 bp to 119316218 bp (shown in green), with the TSS located at 119317130 bp (red); this diagram is based on information from the hg38 assembly of the human genome available via the UCSC genome browser at https://genome-euro.ucsc.edu. Methylation data was obtained from nine probes within the CpG island, five upstream of the TSS (p9-p5) and four downstream (p4-p1)

as indicated. The graphs show methylation levels (M values) in tumour and normal tissue for these nine probes, the genomic position of each probe is also indicated on the graph. The final graph shows aggregate data (the mean M value across the nine probes for tumour and normal tissue samples). The statistical analysis shown here was that obtained using the default setting from the SMART tool (40).

Supplementary Figure 3.

Survival data in breast cancer based on MCAM gene expression. A) Overall survival and B) distant metastasis free survival in HER2+ tumours, TNBC and ER+PR+ tumours stratified for MCAM gene expression. Data in A) and B) is based on microarray platforms. C) Overall survival in all breast cancer types according to MCAM gene expression in 2976 samples as determined by RNA-seq. Data was analysed and displayed using KM plotter (41).

Supplementary Figure 4.

MCAM gene expression in the major cell populations identified in the 26 patient/100064 cell scRNA-seq study of Wu et al (42). This data was obtained and plotted using Single Cell Portal.

Supplementary Figure 5.

Correlations between **A)** sEMT and TGFB1 gene expression and **B)** MCAM and TGFB1 gene expression in 1093 breast cancer patients from TCGA. Spearman's r and 95% confidence intervals (with associated p values) are shown. **C)** Comparison of overall survival of patients in the TCGA cohort based on classification into sEMT groups as indicated. No statistical differences (ns) were found between survival in the three groups using a Mantel-Cox log rank test.

Supplementary Figure 6.

A) Invasion score (sInv) versus cancer stem cell score (sCSC) for sEMT^{low}, sEMT^{med} and sEMT^{high} malignant cells, with position of MCAM^{neg} (grey), MCAM^{low} (green) and MCAM^{high} (red) cells identified.

B) Invasion score (sInv) of sEMT^{low}, sEMT^{med} and sEMT^{high} populations subdivided into MCAM^{neg}, MCAM^{low} and MCAM^{high} cells. Subsets are ordered on the vertical axis based on their mean sInv. A Mann Whitney test indicated that the sEMT^{high}MCAM^{low} population has a significantly greater sInv than any other subset; ****P<0.0001.

C) As in B), except the subsets were scored for sCSC.

Supplementary Figure 7

Survival data for TCGA cohort breast cancer patients according to sEMT classification and MCAM expression. For each group (sEMT^{low}, sEMT^{med} and sEMT^{high}), tumours were classified as either MCAM^{high} or MCAM^{low} according to their expression above or below the median value. Overall survival was compared between these two groups and analysed using the Mantel-Cox log rank test. No statistical differences (ns) were found in survival between MCAM^{high} and MCAM^{low} expressing tumours in the three groups. The left-hand panels shows the distribution of MCAM gene expression in the different sEMT groups relative to the median value (black horizontal line). The right hand panels show the overall survival in MCAM^{low} (dotted lines) and MCAM^{high} (solid line) expressing tumours.

Supplementary Figure 1

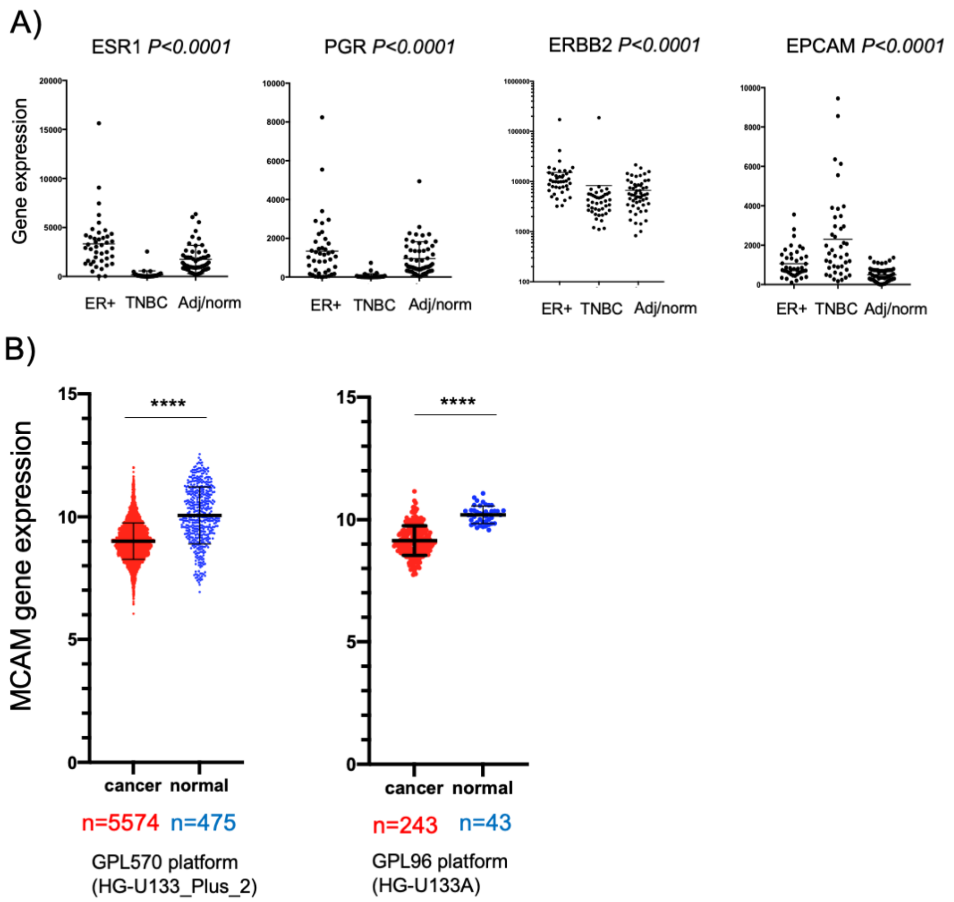

Supplementary Figure 2

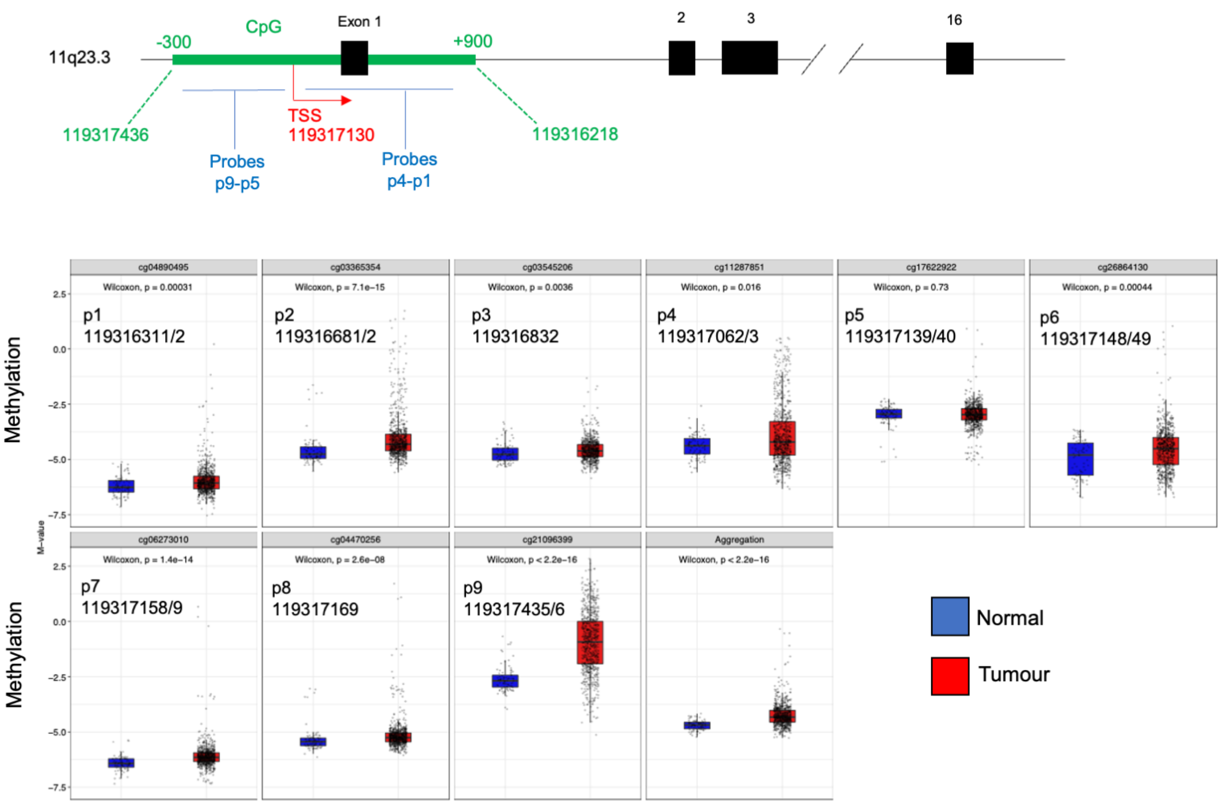

Supplementary Figure 3

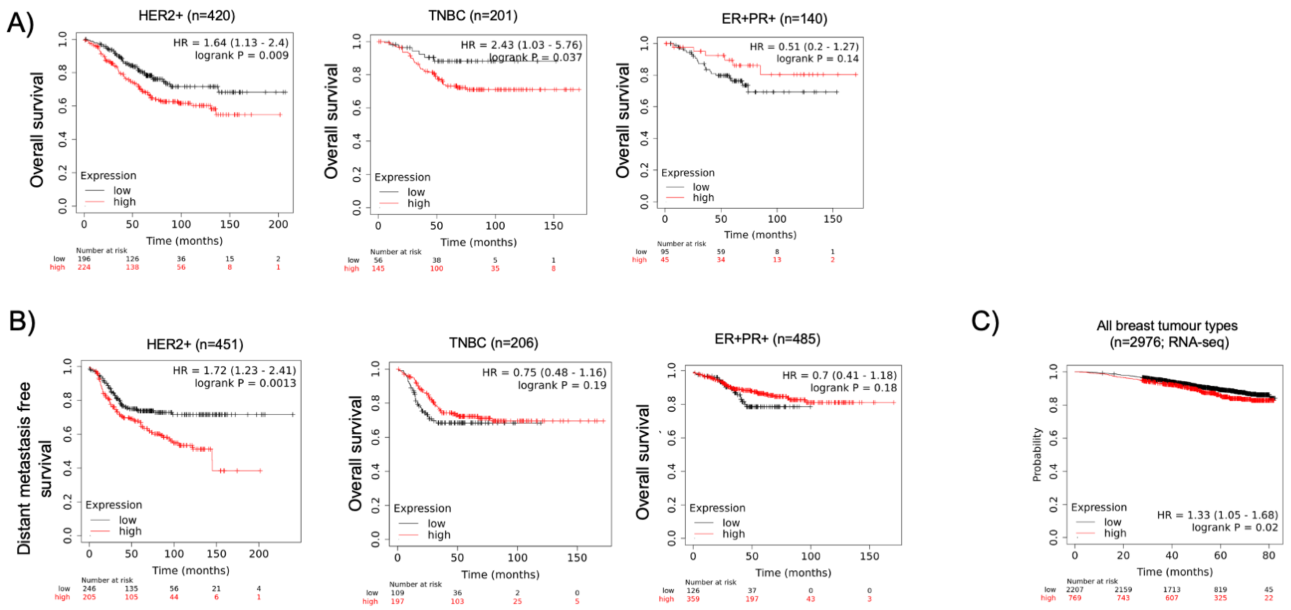

Supplementary Figure 4

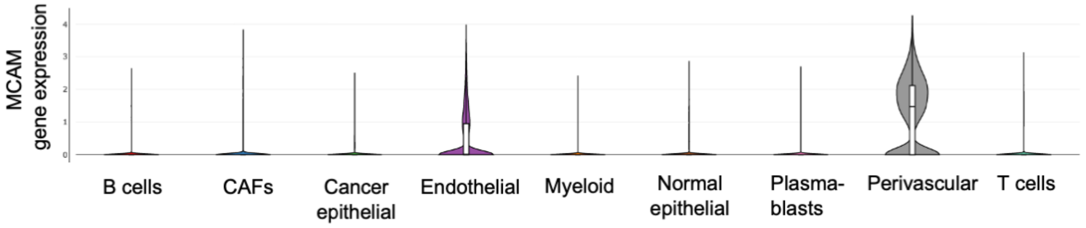

Supplementary Figure 5

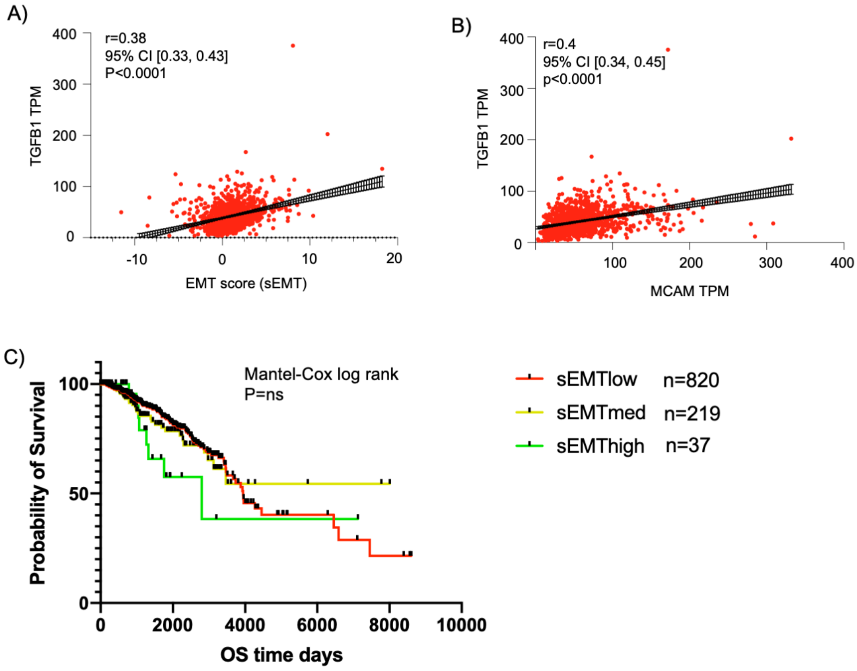

Supplementary Figure 6

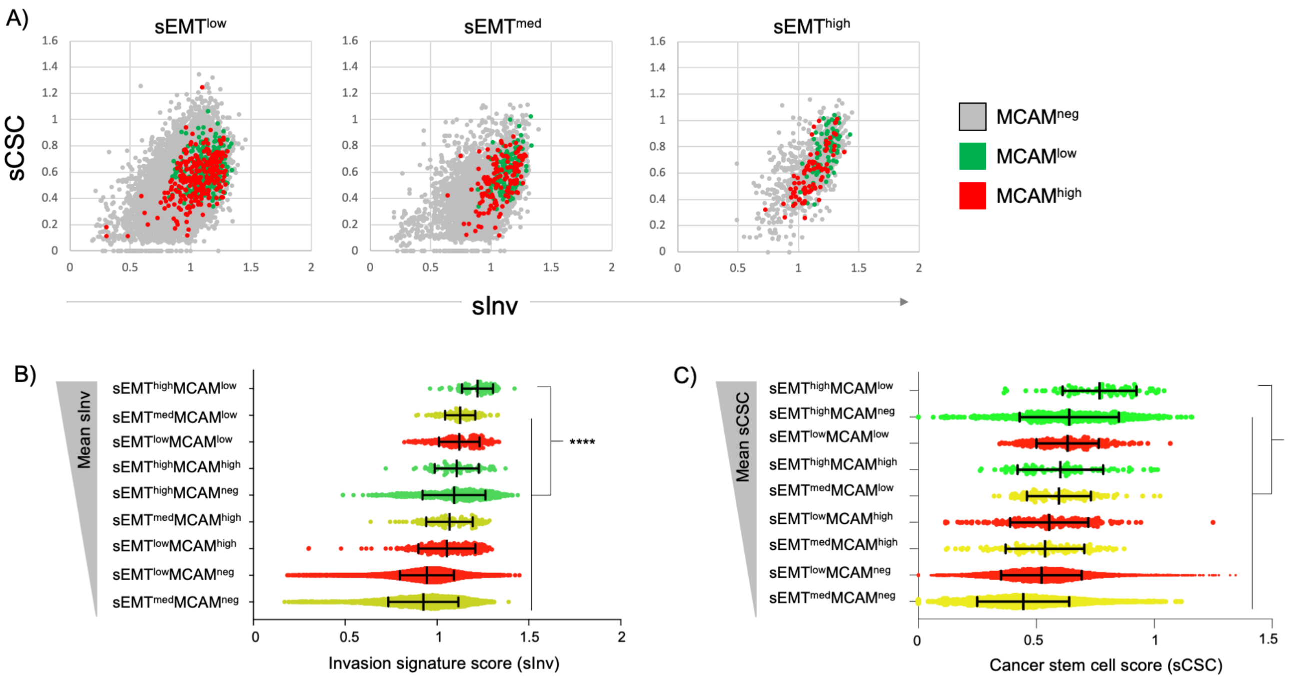

Supplementary Figure 7

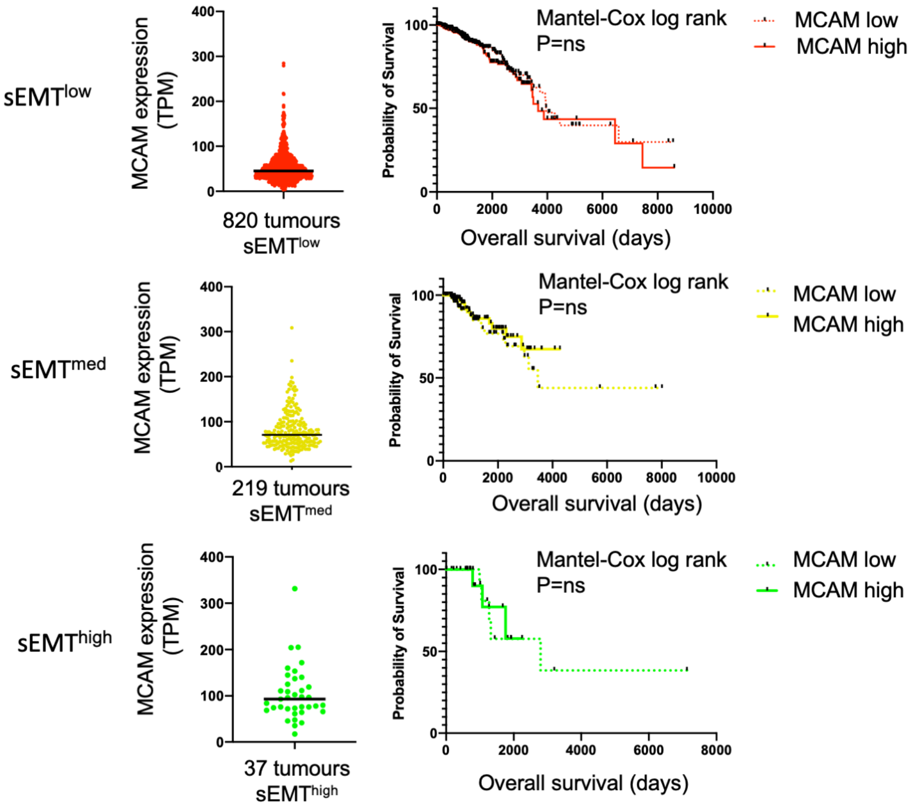

2. Supplementary Tables

Supplementary Tables 1 and 2 are attached in a single excel file

Supplementary Table 1

CD146/MCAM siRNA sequences

Supplementary Table 2

Gene signatures used in this study. The genes used in each signature are listed. The Venn diagram (constructed using https://bioinformatics.psb.ugent.be/webtools/Venn/) shows the degree of overlap of the four signatures; MMP1 appears in both sInv and sCSC. The table includes details of how each score was calculated and references.
